# Is young‐onset esophageal adenocarcinoma increasing in Japan? An analysis of population‐based cancer registries

**DOI:** 10.1002/cam4.4528

**Published:** 2022-01-25

**Authors:** Eiko Saito, Tomonori Yano, Megumi Hori, Daisuke Yoneoka, Tomohiro Matsuda, Yichi Chen, Kota Katanoda

**Affiliations:** ^1^ Division of Cancer Statistics Integration Center for Cancer Control and Information Services National Cancer Center Chuo‐ku Tokyo Japan; ^2^ Department of Gastroenterology and Endoscopy National Cancer Center Hospital East Kashiwa Chiba Japan; ^3^ Division of Biostatistics and Bioinformatics Graduate School of Public Health St. Luke’s International University Tokyo Japan; ^4^ National Cancer Registry Section Center for Cancer Control and Information Services National Cancer Center Chuo‐ku Tokyo Japan; ^5^ Department of Global Health Policy Graduate School of Medicine The University of Tokyo Bunkyo‐ku Tokyo Japan

**Keywords:** age‐period‐cohort model, cancer registry, esophageal cancer, incidence, missing data, multiple imputation, neoplasms

## Abstract

**Background:**

While esophageal squamous cell carcinoma (ESCC) is the predominant histological type in Japan, concern has been expressed over an increase in the proportion of esophageal adenocarcinoma (EAC), especially in middle‐aged populations. This study aimed to assess long‐term trends in esophageal cancer incidence by histological type.

**Methods:**

We used data from three population‐based cancer registries in Japan with 10,642 esophageal cancer cases diagnosed between 1993 and 2014. The multiple imputation approach was used to impute a specific histological type (ESCC, EAC, and others) for cases with “Unknown” or missing status. We calculated the age‐standardized incidence rates by histological type from 1993 to 2014 and fitted age‐period‐cohort models to estimate the annual percent changes (APCs) and adjusted incidence rate ratios (IRRs).

**Results:**

After imputation of missing data, the largest mean APC increase was seen in the incidence of EAC in men aged 40–49 years (7.1%) followed by those aged 50–59 years (5.5%). The age‐period‐cohort analysis showed that men who were born in the 1960s and later were more likely to develop EAC relative to men who were born in 1950–1959 (1960–1969 cohort, IRR: 1.42; 1970–1974 cohort, IRR: 2.23), with a period effect indicating a constant increase after 2003. For women, no significant trend in EAC incidence was observed.

**Conclusions:**

The incidence of EAC has increased more prominently compared with that of ESCC, especially in men aged 40–59 years, suggesting the impact of increasing obesity in men and a reduction in *H*. *pylori* prevalence in Japan.

## Graphical abstract

This is the first study to report that between 1993 and 2014, the incidence of young‐onset esophageal adenocarcinoma increased in middle‐aged men (40–59 years old) in Japan, where squamous cell carcinoma is a predominant subtype of esophageal cancer. This study used high‐quality cancer registries containing histological type with 20 years of diagnosis and more than 50 years of birth cohort before and after World War II.

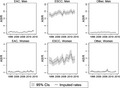

## 1 INTRODUCTION

Esophageal adenocarcinoma (EAC), which is causatively linked primarily with obesity, gastroesophageal reflux disease, and Barrett's esophagus,^1^, ^2^, ^3^ is a major histological type in Western countries. While esophageal squamous cell carcinoma (ESCC) constitutes around 90% of esophageal cancer in Japan, concern has been expressed that the proportion of EAC has increased in recent years.^4^, ^5^ Regarding cause, the rapid transition to a westernized lifestyle followed by an increase in obesity since the late 1950s is suggested to have played a major role; for instance, the National Health and Nutrition Survey, which has followed the lifestyle change in Japanese since 1947, reported that the frequency of overweight and obese adults with a BMI above 25 kg/m^2^ has increased over the past several decades, particularly in men born in the 1970s.^6^, ^7^, ^8^ The impact of rising obesity on the incidence of EAC has already been identified in the United States,^9^ yet its impact remains unknown in Japan. Furthermore, infection with *Helicobacter pylori* (*H*. *pylori*) is protective against gastroesophageal reflux disease (GERD) and subsequent progression to Barrett's esophagus and esophageal adenocarcinoma.^1^ However, the prevalence of *H*. *pylori* infection has dramatically decreased among recent birth cohorts, to as low as 10% for individuals born after 1998.^10^

To elucidate how chronological changes in risk factors impact the occurrence of EAC, a time trend analysis of esophageal cancer incidence by major histological type would be useful. However, the previous under‐reporting of cancer subtypes remains a major barrier to the analysis of cancer registry data in all countries. In recent years, the multiple imputation method has been used to account for missing values. This method yields a mean estimate and standard error to incorporate variance between imputed values.^11^, ^12^, ^13^, ^14^, ^15^ In the present study, we assessed long‐term trends in esophageal cancer incidence by major histological type using population‐based cancer registry data in Japan.

## 2 METHODS

### 2.1 Data sources

We used data from three population‐based cancer registries in Japan (Yamagata, northeastern region; Fukui, Sea of Japan region; Nagasaki, southern region) under the Monitoring of Cancer Incidence in Japan Project.^16^ These registries were established with the aim of monitoring long‐term trends in cancer incidence with a high degree of completeness and timeliness, and the incidence is, therefore, less likely to be affected by changes in data quality.^17^, ^18^ Trends from these prefectures in combination are comparable to those observed in the overall Japanese population.^17^

We extracted 10,642 esophageal cancer cases diagnosed between 1993 and 2014 from these three prefectures. The incidence of esophageal cancer was classified according to the International Statistical Classification of Diseases and Related Health Problems, 10th Revision (ICD‐10) code of “C15.” This study was approved by the Institutional Review Board of the National Cancer Center in Tokyo (approval number 2004‐061).

### 2.2 Imputation method

We grouped histological type into three major categories using the International Classification of Diseases for Oncology, 3^rd^ Edition (ICD‐O‐3) morphology codes, namely esophageal squamous cell carcinoma (ESCC, 8050–8084), esophageal adenocarcinoma (EAC, 8140–8384), and other specified malignant neoplasm (other, 8011–8046, 8090–8131, and 8380–9581). We treated cases with “Unknown” as histological type and neoplasms not otherwise specified (NOS) (8000–8010) as missing this value, and imputed values by the multiple imputation approach. We assumed that the missing cases were not completely randomly distributed among cases (missing completely at random, MCAR), but rather that the missing patterns of histological types depended on the observed data (missing at random, MAR).

The imputation model adapted variables from the cancer registry data that can predict the histological type and its missingness. The model used the following covariates: sex; age at diagnosis; prefecture; year of diagnosis; whether the case was DCN (death certificate notification) or not; whether the case was DCO (death certificate only) or not; clinical stage at diagnosis (localized, regional, or distant); whether the tumor was detected by screening (yes or no); type of primary treatment received, including surgery (yes or no), chemotherapy (yes or no), radiotherapy (yes or no), and endoscopy (yes or no); observation period (from the time of diagnosis to last confirmed year of survival, year of death, or censor year, whichever came first); and the vital status during the observation period. Unless death or the last confirmed date of survival was reported, the observation period was censored at the last year and month of follow‐up (2016 for Yamagata and Fukui and 2015 for Nagasaki). In addition, we used a prefecture‐level Gini coefficient (an index of income inequality), which ranges from 0 (perfect equality) to 1 (perfect inequality),^19^ to adjust for socioeconomic inequalities.^20^, ^21^ The imputation model took each case with a missing value and imputed for it a specific histological type (ESCC, EAC, or other types). Multiple imputation with chained equations was performed to impute each variable with a missing value using the remaining variables in the dataset so that no variable was left with a missing observation.^14^, ^15^, ^22^ This imputation method has been described in detail in a previous study on prostate cancer incidence.^15^ Validity of the imputation model was assessed using the Kaplan–Meier log‐rank test of equality with Bonferroni correction for overall survival by histological type and by year of diagnosis, comparing the complete dataset excluding the missing values and 10 imputed datasets^12^: if the imputation is valid, survival probability by each histological type obtained from the imputed datasets should not be significantly different from the complete dataset, because the prognosis differs by histological type.^12^

### 2.3 Trend analysis

For the trend analyses, we calculated the age‐standardized incidence rates (ASIRs) by histological type and sex from 1993 to 2014 using a 1985 Japanese standard population for each of the imputed datasets. We then took an average of the imputed rates to estimate a single ASIR along with its 95% CIs. Furthermore, a Poisson age‐period‐cohort model as described by Rutherford et al.^23^ was fitted to estimate the annual percent change (APC) per calendar year for ESCC and EAC in men and women, respectively. To obtain stable parameter estimates across histological types, 10‐year intervals were used to categorize age groups above 40 years (i.e., 40–49, 50–59, 60–69, 70–79, 80+), and 10‐year intervals to categorize birth cohort (i.e., 1989–1909, 1910–1919, 1920–1929…, 1970–1974). We estimated adjusted incidence rate ratios (IRRs) and 95% CIs to compare incidence rates for a given birth cohort relative to people in the 1950–1959 birth cohort. The same procedure was repeated to estimate the IRRs for cases diagnosed in a given calendar year relative to the cases diagnosed in the calendar year 2000. We arbitrarily chose the 1950–1959 birth cohort as reference for cohort effect, and 2000 as reference for period effect, taking the approximate midpoints in birth cohorts or the year of diagnosis. We further assessed the APC and adjusted IRRs by clinical stage at diagnosis for each histological type. Analysis of EAC incidence in women was restricted to those aged 60 years and older to ensure a sufficient number of cases. We performed additional sensitivity analyses with a reference cohort sourced from within the 1940–1949 birth cohort. Furthermore, the same analyses as those used to assess period and cohort effects on esophageal cancer incidence were conducted using original data before imputation but excluding the “Unknown” histological type. We also assessed trends in smoking rates in these three prefectures between 2000 and 2019.^24^ All analyses were conducted using STATA version 15.0 software (StataCorp LP), and *p* < 0.05 was considered to denote statistical significance.

## 3 RESULTS

Table 1 shows the distribution of esophageal cancer cases by reported histological type, including unknown and missing status for each of the selected covariates (also shown in Table S1 by reported histological type, sex, and age group). Patients with unknown/missing morphology were likely to be older and the year of diagnosis tended to be earlier than patients with a reported histological type. Patients with less than 5 years of observation had a higher rate of unknown/missing data for histological type than those with a longer observation period. Table S2 compares the Kaplan–Meier log‐rank test for equality of survival probability by histological type and by year of diagnosis between the complete dataset and imputed datasets. Estimated survival probability did not differ significantly across sex and histological types.

**TABLE 1:** Distribution of esophageal cancer cases by histological type and selected covariates, 1993–2014

	Total number of cases	Histological type (%)			
		Squamous cell carcinoma	Adeno carcinoma	Other types	Unknown/missing
All cases	10,642	82.6	4.0	2.2	11.2
Age at diagnosis					
<50 years	336	78.3	7.4	3.9	10.4
50–<60 years	1580	86.5	4.3	2.6	6.7
60–<70 years	3425	86.8	3.7	2.3	7.3
70–<80 years	3478	84.3	3.5	2.2	10.0
80+ years	1823	68.9	4.9	1.3	24.9
Prefecture					
Yamagata	4710	81.1	4.1	1.9	12.9
Fukui	1693	81.3	4.7	1.8	12.2
Nagasaki	4239	84.7	3.6	2.7	9.0
Year of diagnosis					
1993 to <2000	2717	80.1	3.9	1.9	14.1
2000 to <2005	2382	79.6	2.5	2.3	15.6
2005 to <2010	2626	84.1	3.4	3.2	9.3
2010 to <2015	2917	86.0	5.9	1.4	6.7
Death certificate notification					
Yes	1086	32.4	1.7	1.5	64.5
No	9556	88.3	4.3	2.3	5.2
Clinical stage at diagnosis					
Localized	3126	90.2	5.4	2.0	2.4
Regional	3704	90.7	3.1	2.1	4.0
Distant	1610	82.6	5.6	3.6	8.3
Unknown/missing	2202	58.1	2.3	1.6	38.0
Observation period					
0 to <5 years	8120	80.4	3.9	2.2	13.5
5 to <10 years	1744	89.8	4.5	2.2	3.5
10 to <15 years	520	89.6	4.4	2.1	3.9
15+ years	258	89.9	3.1	1.6	5.4
Screening detected					
Yes	9608	84.9	4.1	2.2	8.7
No	684	90.4	4.4	2.8	2.5
Unknown/missing	350	3.4	0.0	0.0	96.6
Surgery					
Yes	3272	89.5	4.8	3.0	2.6
No	6362	86.6	4.0	1.9	7.6
Unknown/missing	1008	35.0	1.7	1.2	62.1
Chemotherapy					
Yes	3953	90.4	3.4	2.7	3.5
No	5648	85.2	4.9	2.1	7.8
Unknown/missing	1041	38.5	1.5	1.0	59.0
Radiotherapy					
Yes	3708	91.9	1.8	1.8	4.5
No	5887	84.6	5.8	2.6	7.0
Unknown/missing	1047	38.3	1.9	1.1	58.7
Endoscopy					
Yes	1288	89.1	6.6	1.2	3.2
No	8147	87.3	3.9	2.5	6.4
Unknown/missing	1207	44.2	2.2	1.2	52.4
Gini coefficient					
Mean	10,642	0.321	0.326	0.318	0.311

### 3.1 Age‐standardized incidence rates and annual percent changes

**FIGURE 1:**
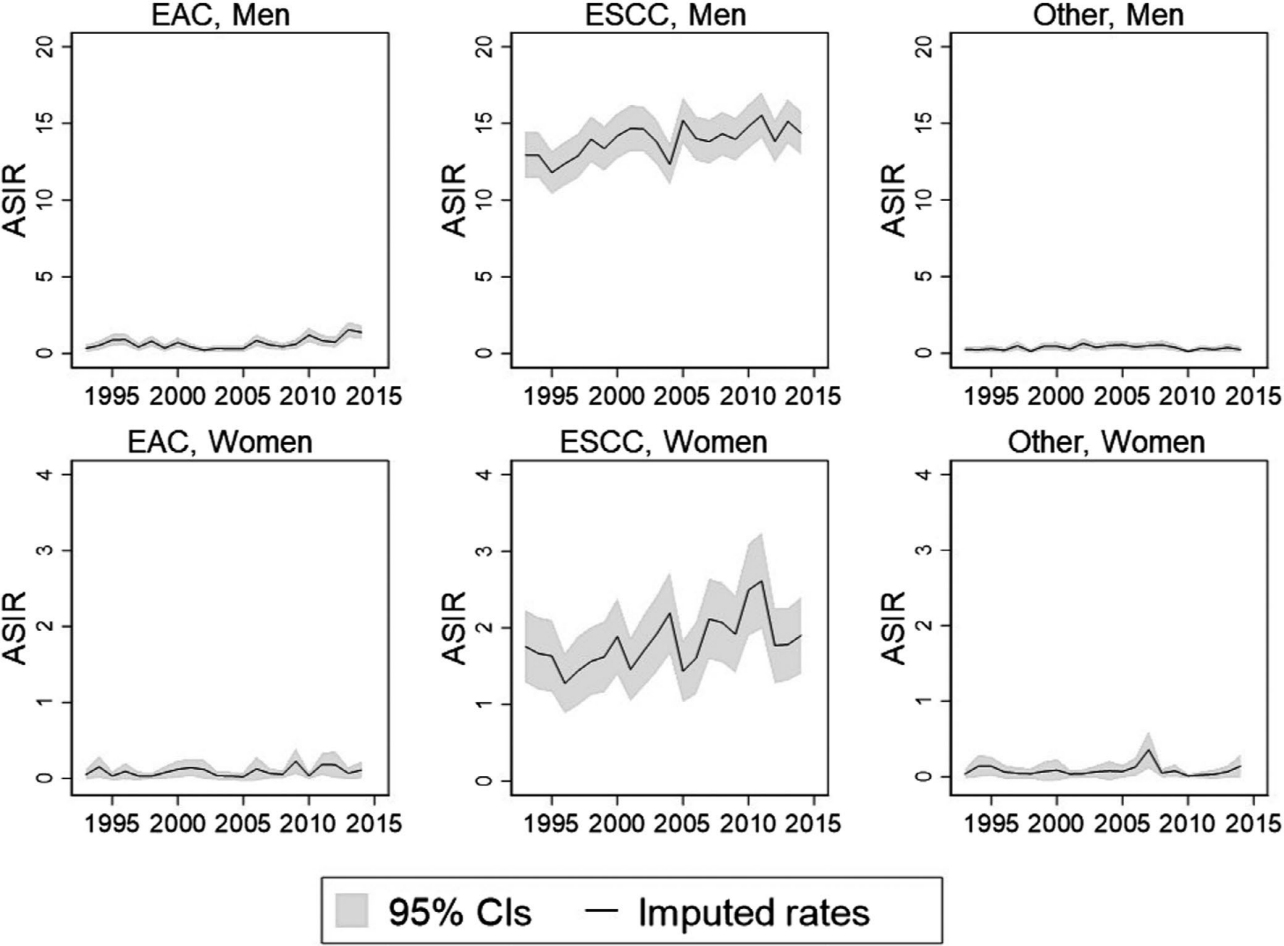
Age‐standardized incidence rates of esophageal cancer after imputation of missing data, 1993–2014

Figure 1 shows the age‐standardized incidence rates of esophageal cancer by histological type and by sex after imputation of missing data. Overall, the age‐standardized incidence rates were higher for men than women across all histological types. After imputation of missing data, a constant increase was seen in the ASIR of EAC from 1993 (0.3 per 100,000) to 2014 (1.4 per 100,000) in men. The age‐adjusted incidence rates of ESCC in men also showed a slight increase from 1993 (13.0 per 100,000) to 2014 (14.4 per 100,000) with higher variability than other histological types. In contrast, no pronounced change was seen in the incidence rates of other types in men, or across all histological types in women.

After fitting the age‐period‐cohort model to those over 40 years old, the largest mean APC increase was seen in the incidence of male EAC in those aged 40–49 years (7.1%), followed by those aged 50–59 years (5.5%). For ESCC, a minor increase in mean APC between 1993 and 2014 was seen in men above 50 years old (0.3%–1.0%) (Table 2). The incidence of ESCC in younger women below 60 years showed a slight increase (0.8%–1.5%) but no increase was seen in those above 70 years old. The incidence of EAC in women showed a constant decrease in those above 70 years old. Table S3 shows the APC by clinical stage at diagnosis for each histological type in men and women. The increase in mean APC was more pronounced for localized EAC and ESCC than more advanced stages in men, while that in women was inconclusive due to large confidence intervals.

**TABLE 2:** Mean annual percent change in the incidence rates of esophageal cancer by histological type, 1993–2014

Age group	EAC		ESCC	
	Mean APC (%)	95% CI	Mean APC (%)	95% CI
Men				
40–49	7.09	(6.09, 8.86)	−1.03	(−1.10, −0.96)
50–59	5.52	(4.90, 6.31)	0.25	(0.25, 0.25)
60–69	3.81	(3.58, 4.06)	0.96	(0.96, 0.96)
70–79	1.82	(1.58, 2.09)	0.82	(0.82, 0.83)
80+	4.99	(3.81, 6.61)	1.01	(0.99, 1.03)
Women				
40–49	(Omitted)^a^		0.81	(0.56, 1.01)
50–59	(Omitted)^a^		1.78	(1.69, 1.88)
60–69	0.90	(−0.06, 5.53)	1.45	(1.42, 1.48)
70–79	−0.96	(−1.02, −0.65)	−1.19	(−1.19, −1.20)
80+	−3.06	(−3.28, −2.72)	−2.54	(−2.57, −2.50)

Abbreviations: APC, annual percent change; EAC, esophageal adenocarcinoma; ESCC, esophageal squamous cell carcinoma.

^a^ Omitted because of limited cases.

### 3.2 Period and cohort effects

**FIGURE 2:**
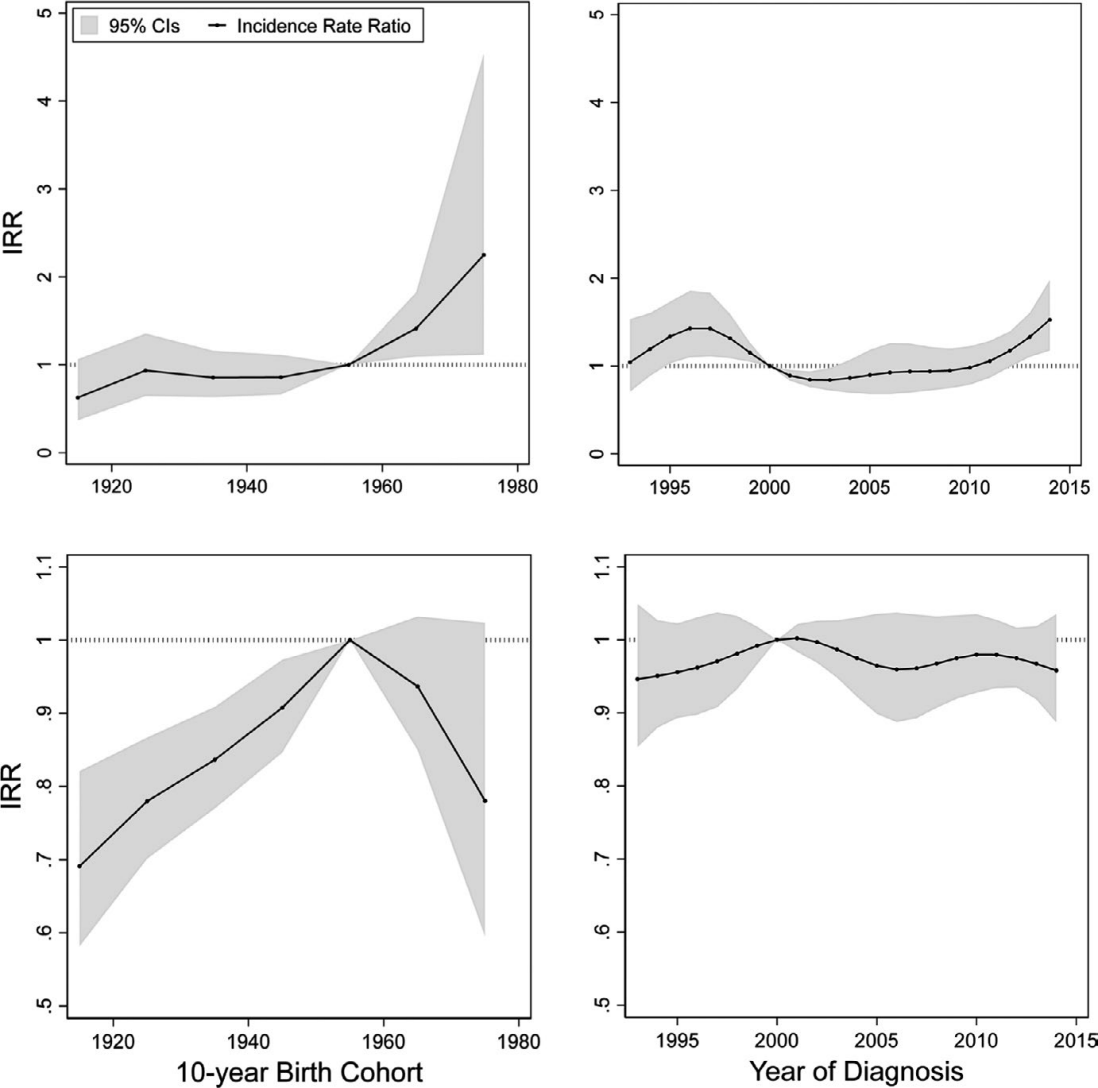
Parameter estimates of cohort and period effects for esophageal cancer incidence in men by histological type

**FIGURE 3:**
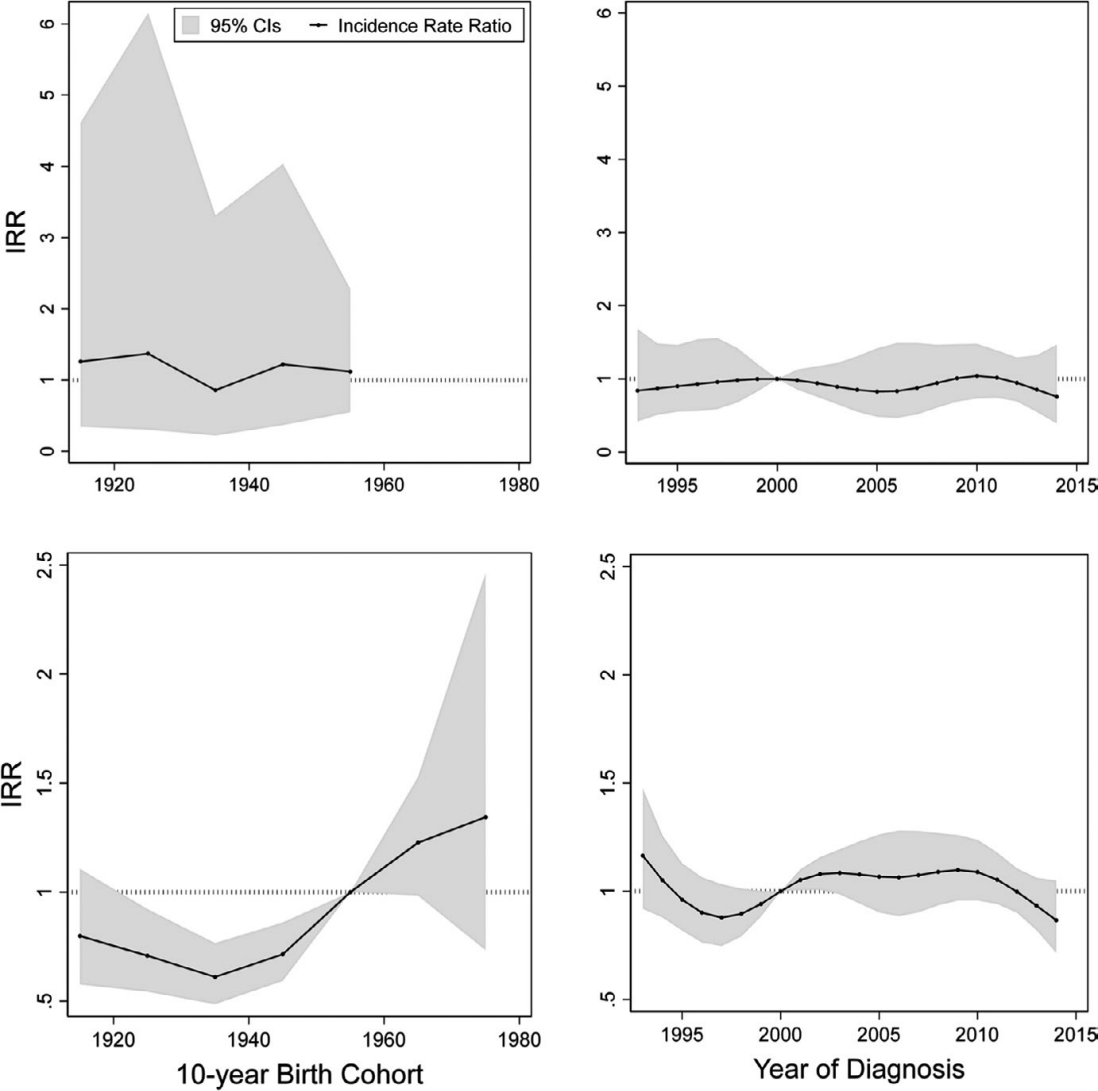
Parameter estimates of period and cohort effects for esophageal cancer incidence in women by histological type

Figure 2 shows the cohort effects for the IRRs of EAC and ESCC in men after adjusting for age and period effects, and the period effects for the IRRs after adjusting for age and cohort effects. Men born in the 1960s and later were more likely to develop EAC relative to men born in 1950–59 (1960–1969 cohort, IRR: 1.42; 1970–1974 cohort, IRR: 2.23). After adjusting for cohort effect, the incidence of EAC showed a steady increase after 2005, which resulted in years 2013–2014 showing significantly higher IRRs relative to the year 2000 (2013, IRR: 1.23, 2014: 1.39). As for ESCC, birth cohorts before 1950 showed significantly lower IRRs relative to men born in 1950–1959, while no clear period effect was observed for the incidence of ESCC in men. Figure 3 shows the IRRs of EAC (60 years and above) and ESCC in women aged over 40 years. No significant period effect was seen in the IRRs of either EAC or ESCC during the study period. However, women who were born before 1950 showed a significantly lower incidence of ESCC compared to women born during 1950–1959. Figures 1, 2, 3 show the IRRs of EAC and ESCC, respectively. Localized EAC showed higher IRRs in men born in the 1960s and later yet with wide confidence intervals, while more advanced EAC showed no clear trend. Localized ESCC in men showed a steady increase in IRRs in more recent birth cohorts, while those of regional and distant ESCC showed a decrease in cohorts born in the 1960s and later. When we changed the reference cohort to those born in the 1940s instead of the 1950s, men born in the 1950s showed significantly higher ESCC incidence than those born in the 1940s (Figure S4). Furthermore, ESCC incidence in women aged 60 years and above was significantly higher in those born in the 1950s and later relative to women born in the 1940s. Our sensitivity analysis using original data before imputation, with the exclusion of the “Unknown” histological type, showed wider fluctuations in period and cohort effects on incidence in male EAC in earlier years but did not show substantial change from the results obtained from the imputed data (Figures S5 and S6). Furthermore, trends in smoking prevalence in these three prefectures were similar to those in the overall Japanese population (Figure S7).

## 4 DISCUSSION

This is the first study to identify long‐term trends in esophageal cancer incidence by subtype, disaggregated by period effects and cohort effects over 20 years of diagnosis and more than 50 years of birth cohort before and after World War II. This is also the first study to report that between 1993 and 2014, the overall incidence of EAC, especially of localized EAC, increased in middle‐aged men (40–59 years old) in Japan. Although previous studies from Osaka (West Japan) and Akita (Northeast Japan) prefectures reported a general increase in EAC incidence,^4^, ^25^ they did not analyze recent age‐specific trends. Moreover, a recent study analyzed trends in esophageal cancer of all types only, and not by subtype.^26^

When we decomposed the observed changes into cohort effects with adjustment for period and age effects, men born in the 1960s and later had a significantly higher risk of developing EAC relative to men who were born in 1950–1959. This observed birth cohort effect might be explained by the fact that incidence of *H*. *pylori* infection in Japan has seen a marked and rapid decline, from around 70% in Japanese born before 1950 to nearly 5% in those born after 2000,^10^, ^27^ primarily due to improvements in hygiene and sanitation, which affect the likelihood of *H*. *pylori* infection during childhood. Given that *H*. *pylori* infection is an established protective factor against GERD, Barrett's esophagus, and subsequent EAC via decreased acid secretion,^1^, ^3^ the increase in EAC incidence in those born from 1960 may be associated with the decrease in *H*. *pylori* prevalence since the 1950s.

Our study also showed a significant period effect after 2013, namely an increase in EAC incidence relative to the year 2000 with adjustment for cohort and age effects. This period increase in EAC incidence among men coincides with the initiation in February 2013 of insurance coverage under the national health insurance scheme for eradication therapy for chronic gastritis (including asymptomatic patients) due to *H*. *pylori* infection.^28^ A recent meta‐analysis reported that successful eradication of *H. pylori* significantly increases the risk of reflux esophagitis irrespective of BMI,^29^, ^30^ possibly by inducing a resumption in acid production capacity.^31^ However, its immediate impact on the development of EAC has not been clarified, and it may be too early to conclude a link between eradication therapy and the observed increase in EAC. Furthermore, an upward change in the IRRs of EAC in men during the past decade may also be explained by an increase in obesity––for instance, the proportion of overweight and obese adults (BMI ≥25) nearly doubled over the past few decades in men across age groups, which could be inferred as a period effect.^32^ The prevalence of obesity in the three prefectures was comparable to the national prevalence of obesity.^33^ Increases in the early onset of obesity‐related cancers have been reported in other countries: for instance, an increasing incidence of esophageal adenocarcinoma has been observed in US adults starting at age 50 years.^34^ On the contrary, the level of obesity did not change in Japanese women over time, with a prevalence of overweight and obese adults at 20.7% in 1980 versus 21.9% in 2017.^32^ This may explain why no significant trends were seen in female EAC incidence.

Notably, men who were born before 1950 had lower IRRs of ESCC relative to men born between 1950 and 1959. IRRs then started to decline again in cohorts born in the 1960s and later. The main risk factors for ESCC are smoking and alcohol drinking, which differ from those for EAC.^35^ As such, one way to explain this inverted U‐shaped trend with a peak in the 1950s birth cohort may be differences in smoking prevalence by birth cohort. Smoking prevalence in Japanese men peaked in the late 1950s birth cohort^36^ and started to decrease in cohorts born in the 1960s and later,^37^ which concurs with our study findings. The overall increase in ESCC incidence in Japanese men and women aged less than 70 years between 1993 and 2014 is also consistent with findings from a previous study^38^ and might be due to the increased use of endoscopy and improved accuracy in the cancer registration system.

Although women born in the 1950s and later had higher IRRS of ESCC than those born before 1950, the overall incidence of ESCC in Japanese women remained considerably low (around 2 per 100,000 on average), possibly due to low prevalence of smoking (current smokers: 8.4% in women vs. 32.2% in men as of 2010) and drinking (regular drinkers: 6.9% in women vs. 35.4% in men as of 2010),^33^ and no significant period effects were detected after adjusting for age and cohort effects.

Our results reaffirm the importance and necessity of careful endoscopic inspection of the upper gastrointestinal tract. The Japanese Guidelines for Gastric Cancer Screening currently recommend endoscopic screening in those aged 50 years and over, with an interval of 2 or 3 years,^39^ and 42.9% of those aged 50–69 years have in fact undergone gastric cancer screening (including both x‐ray and endoscopy) as of 2019.^24^ With the rise in young‐onset localized EAC in recent birth cohorts, closer attention should be paid to endoscopic inspection of the whole upper gastrointestinal tract in younger generations in conjunction with gastric cancer screening. Moreover, physicians should modify the endoscopic surveillance method to cover the gastroesophageal junction more carefully, particularly in middle‐aged subjects without findings of *H*. *pylori* infection. Furthermore, since the majority of esophageal cancer incidence is attributable to smoking and drinking,^40^ interventions aiming at reducing tobacco smoking and drinking may have an impact on controlling the occurrence of esophageal cancer.

The strength of this study lies in its use of high‐quality, population‐based cancer registry data over time in a Japanese population, while ensuring comparability with the national estimates of cancer incidence. Moreover, the three cancer registries used are geographically distributed, from the Tohoku (northeastern Japan) to Kyushu (southern Japan) regions, minimizing the possibility of geographic bias.

Some limitations warrant mention. First, the completeness of histology reporting has fluctuated over time, with the proportion of cases with an unknown or missing status for histological type remaining at 14.1% in 1993–1999 and 15.6% in 2000–2004, but decreasing to 6.7% in 2010–2014. A decrease in the proportion of unknown or missing cases and the resulting increase in the share of reported histological types may have induced bias in the incidence trends. However, we imputed histological type using the multiple imputation approach, and validated the imputation results comparing survival probability by histological type between the complete dataset and imputed datasets. Because the survival probability (i.e., prognosis) differs between ESCC and EAC, the imputed dataset should yield a survival probability similar to the original data for each of the histological types. Our validation results showed that the survival probability did not differ between the original and imputed data, meaning that the imputation model correctly assigned histological types to each patient, although we cannot exclude the possibility of residual bias induced by other unknown factors. Furthermore, our sensitivity analysis using original data excluding unknown and missing histological types did not show substantial change from the results obtained from the imputed data. Second, the quality of cancer incidence data obtained from these three high‐quality registries has been stable over time, yet the incidence of esophageal cancer may have deviated from that of other cancer registries in Japan. Against this, however, the incidence and trend estimations are representative of those for the overall Japanese population from a previous study.^17^ Finally, the increase in young‐onset EAC might arguably be due to improved accuracy of diagnostic and screening practices with endoscopy and cancer registration, particularly after 2002, when the Ministry of Health, Labour, and Welfare started designating cancer hospitals, which had the effect of also promoting hospital‐based cancer registration.^41^ However, the cohort effects shown in our study are after adjustment of period effects, which minimizes any bias induced by such systematic yet momentary changes.

In conclusion, the incidence of EAC has increased more prominently compared with that of ESCC, particularly in men aged 40–59 years. This recent period increase in EAC incidence in men born from 1960 may be associated with a decrease in *H*. *pylori* prevalence coupled with an increase in obesity over time.

## CONFLICT OF INTEREST

None declared.

## AUTHOR CONTRIBUTIONS

Eiko Saito analyzed the data, drafted the manuscript, reviewed and edited the manuscript, and contributed to discussion; Tomonori Yano and Kota Katanoda designed and supervised the study, reviewed and edited the manuscript, and contributed to discussion; Megumi Hori and Daisuke Yoneoka supervised the statistical analysis, reviewed and edited the manuscript, and contributed to discussion; and Tomohiro Matsuda and Yichi Chen reviewed and edited the manuscript, and contributed to the discussion. All authors read and approved the final manuscript.

## ETHICAL APPROVAL STATEMENT

This study was approved by the Institutional Review Board of the National Cancer Center in Tokyo (approval number 2004‐061).

## Supporting information

Supplementary Material

## Data Availability

Regarding data accessibility, we cannot publicly share the individual data even after anonymization according to the data use rules in Japan. Currently, only investigators who fulfill the requirements of conducting research projects are eligible to use the MCIJ data. The investigators are required to submit a Project Protocol (research question, aim, background, design, and analytical plan) with the consent of IRB for review by the committees in the local governments (prefectures). Requests can be made by contacting the prefectures directly.

## References

[1] Lagergren J . Adenocarcinoma of the oesophagus: what exactly is the size of the problem and who is at risk? Gut. 2005;54(Suppl 1):i1‐i5. 15711002 10.1136/gut.2004.041517 PMC1867797

[2] Kubo A , Corley DA . Body mass index and adenocarcinomas of the esophagus or gastric cardia: a systematic review and meta‐analysis. Cancer Epidemiol Biomarkers Prev. 2006;15(5):872‐878. 16702363 10.1158/1055-9965.EPI-05-0860

[3] Coleman HG , Xie SH , Lagergren J . The epidemiology of esophageal adenocarcinoma. Gastroenterology. 2018;154(2):390‐405. 28780073 10.1053/j.gastro.2017.07.046

[4] Koizumi S , Motoyama S , Iijima K . Is the incidence of esophageal adenocarcinoma increasing in Japan? Trends from the data of a hospital‐based registration system in Akita Prefecture, Japan. J Gastroenterol. 2018;53(7):827‐833. 29134330 10.1007/s00535-017-1412-4

[5] Kusano C , Gotoda T , Khor CJ , et al. Changing trends in the proportion of adenocarcinoma of the esophagogastric junction in a large tertiary referral center in Japan. J Gastroenterol Hepatol. 2008;23(11):1662‐1665. 19120859 10.1111/j.1440-1746.2008.05572.x

[6] Nishi N . Monitoring obesity trends in health Japan 21. J Nutr Sci Vitaminol. 2015;61(Suppl):S17‐S19. 26598843 10.3177/jnsv.61.S17

[7] Tarui I , Okada E , Okada C , Saito A , Takimoto H . Trends in BMI among elderly Japanese population: findings from 1973 to 2016 Japan National Health and Nutrition Survey. Public Health Nutr. 2020;23(11):1907‐1915. 32513347 10.1017/S1368980019004828 PMC10200467

[8] Okui T . An age‐period‐cohort analysis of biomarkers of lifestyle‐related diseases using the National Health and Nutrition Survey in Japan, 1973–2018. Int J Environ Res Public Health. 2020;17(21):1973‐2018. 10.3390/ijerph17218159 PMC7663829 33158284

[9] Codipilly DC , Sawas T , Dhaliwal L , et al. Epidemiology and outcomes of young‐onset esophageal adenocarcinoma: an analysis from a population‐based database. Cancer Epidemiol Biomarkers Prev. 2021;30(1):142‐149. 33328255 10.1158/1055-9965.EPI-20-0944 PMC7855414

[10] Wang C , Nishiyama T , Kikuchi S , et al. Changing trends in the prevalence of *H. pylori* infection in Japan (1908–2003): a systematic review and meta‐regression analysis of 170,752 individuals. Sci Rep. 2017;7(1):15491. 29138514 10.1038/s41598-017-15490-7 PMC5686167

[11] Yu M , Feuer EJ , Cronin KA , Caporaso NE . Use of multiple imputation to correct for bias in lung cancer incidence trends by histologic subtype. Cancer Epidemiol Biomarkers Prev. 2014;23(8):1546‐1558. 24855099 10.1158/1055-9965.EPI-14-0130 PMC4119525

[12] Luo Q , Egger S , Yu XQ , Smith DP , O'Connell DL . Validity of using multiple imputation for "unknown" stage at diagnosis in population‐based cancer registry data. PLoS One. 2017;12(6):e0180033. 28654653 10.1371/journal.pone.0180033 PMC5487067

[13] Eisemann N , Waldmann A , Katalinic A . Imputation of missing values of tumour stage in population‐based cancer registration. BMC Med Res Methodol. 2011;11:129. 21929796 10.1186/1471-2288-11-129 PMC3184281

[14] Howlader N , Noone AM , Yu M , Cronin KA . Use of imputed population‐based cancer registry data as a method of accounting for missing information: application to estrogen receptor status for breast cancer. Am J Epidemiol. 2012;176(4):347‐356. 22842721 10.1093/aje/kwr512 PMC3491971

[15] Saito E , Hori M , Matsuda T , Yoneoka D , Ito Y , Katanoda K . Long‐term trends in prostate cancer incidence by stage at diagnosis in Japan using the multiple imputation approach, 1993–2014. Cancer Epidemiol Biomarkers Prev. 2020;29(6):1222‐1228. 32169995 10.1158/1055-9965.EPI-19-1228

[16] Hori M , Matsuda T , Shibata A , Katanoda K , Sobue T , Nishimoto H . Cancer incidence and incidence rates in Japan in 2009: a study of 32 population‐based cancer registries for the Monitoring of Cancer Incidence in Japan (MCIJ) project. Jpn J Clin Oncol. 2015;45(9):884‐891. 26142437 10.1093/jjco/hyv088

[17] Katanoda K , Ajiki W , Matsuda T , et al. Trend analysis of cancer incidence in Japan using data from selected population‐based cancer registries. Cancer Sci. 2012;103(2):360‐368. 22066698 10.1111/j.1349-7006.2011.02145.x

[18] Katanoda K , Hori M , Matsuda T , et al. An updated report on the trends in cancer incidence and mortality in Japan, 1958–2013. Jpn J Clin Oncol. 2015;45(4):390‐401. 25637502 10.1093/jjco/hyv002

[19] Gastwirth JL . The estimation of the Lorenz curve and Gini index. Rev Econ Stat. 1972;54(3):306‐316.

[20] Kuwahara A , Takachi R , Tsubono Y , Sasazuki S , Inoue M , Tsugane S . Socioeconomic status and gastric cancer survival in Japan. Gastric Cancer. 2010;13(4):222‐230. 21128057 10.1007/s10120-010-0561-4

[21] Ito Y , Nakaya T , Nakayama T , et al. Socioeconomic inequalities in cancer survival: a population‐based study of adult patients diagnosed in Osaka, Japan, during the period 1993–2004. Acta Oncol. 2014;53(10):1423‐1433. 24865119 10.3109/0284186X.2014.912350

[22] Azur MJ , Stuart EA , Frangakis C , Leaf PJ . Multiple imputation by chained equations: what is it and how does it work? Int J Methods Psychiatric Res. 2011;20(1):40‐49. 10.1002/mpr.329 PMC3074241 21499542

[23] Rutherford MJ , Lambert PC , Thompson JR . Age–period–cohort modeling. Stata J. 2010;10(4):606‐627.

[24] National Cancer Center . Cancer Registry and Statistics, Cancer Information Service. 2021. Accessed February 26, 2021. https://ganjoho.jp/reg_stat/statistics/dl_screening/index.html#a16

[25] Matsuno K , Ishihara R , Ohmori M , et al. Time trends in the incidence of esophageal adenocarcinoma, gastric adenocarcinoma, and superficial esophagogastric junction adenocarcinoma. J Gastroenterol. 2019;54(9):784‐791. 30927083 10.1007/s00535-019-01577-7

[26] Katanoda K , Hori M , Saito E , et al. Updated trends in cancer in Japan: incidence in 1985–2015 and mortality in 1958–2018—a sign of a decrease in cancer incidence. J Epidemiol. 2021;31(7):426‐450. 33551387 10.2188/jea.JE20200416 PMC8187612

[27] Inoue M . Changing epidemiology of *Helicobacter pylori* in Japan. Gastric Cancer. 2017;20(Suppl 1):3‐7. 27757699 10.1007/s10120-016-0658-5

[28] Asaka M , Kato M , Sakamoto N . Roadmap to eliminate gastric cancer with *Helicobacter pylori* eradication and consecutive surveillance in Japan. J Gastroenterol. 2014;49(1):1‐8. 24162382 10.1007/s00535-013-0897-8 PMC3895201

[29] Sugimoto M , Murata M , Mizuno H , et al. Endoscopic reflux esophagitis and reflux‐related symptoms after *Helicobacter pylori* eradication therapy: meta‐analysis. J Clin Med. 2020;9(9):3007. 10.3390/jcm9093007 PMC7564218 32961949

[30] Fallone CA , Barkun AN , Friedman G , et al. Is *Helicobacter pylori* eradication associated with gastroesophageal reflux disease? Am J Gastroenterol. 2000;95(4):914‐920. 10763937 10.1111/j.1572-0241.2000.01929.x

[31] Koike T , Ohara S , Sekine H , et al. Increased gastric acid secretion after *Helicobacter pylori* eradication may be a factor for developing reflux oesophagitis. Aliment Pharmacol Ther. 2001;15(6):813‐820. 11380319 10.1046/j.1365-2036.2001.00988.x

[32] Ministry of Health, Labour and Welfare of Japan . National Health and Nutrition Survey. Ministry of Health, Labour and Welfare of Japan; 2017.

[33] Ministry of Health, Labour and Welfare of Japan . National Health and Nutrition Survey. Ministry of Health, Labour and Welfare of Japan; 2010.

[34] Sung H , Siegel RL , Rosenberg PS , Jemal A . Emerging cancer trends among young adults in the USA: analysis of a population‐based cancer registry. Lancet Public Health. 2019;4(3):e137‐e147. 30733056 10.1016/S2468-2667(18)30267-6

[35] Cui RI , Kamatani Y , Takahashi A , et al. Functional variants in ADH1B and ALDH2 coupled with alcohol and smoking synergistically enhance esophageal cancer risk. Gastroenterology. 2009;137(5):1768‐1775. 19698717 10.1053/j.gastro.2009.07.070

[36] Marugame T , Kamo K‐I , Sobue T , et al. Trends in smoking by birth cohorts born between 1900 and 1977 in Japan. Prev Med. 2006;42(2):120‐127. 16271753 10.1016/j.ypmed.2005.09.009

[37] Funatogawa I , Funatogawa T , Yano E . Trends in smoking and lung cancer mortality in Japan, by birth cohort, 1949–2010. Bull World Health Organ. 2013;91(5):332‐340. 23678196 10.2471/BLT.12.108092 PMC3646352

[38] Wang QL , Xie SH , Wahlin K , Lagergren J . Global time trends in the incidence of esophageal squamous cell carcinoma. Clin Epidemiol. 2018;10:717‐728. 29950901 10.2147/CLEP.S166078 PMC6016013

[39] Hamashima C . Update version of the Japanese guidelines for gastric cancer screening. Jpn J Clin Oncol. 2018;48(7):673‐683. 29889263 10.1093/jjco/hyy077

[40] Inoue M , Sawada N , Matsuda T , et al. Attributable causes of cancer in Japan in 2005–systematic assessment to estimate current burden of cancer attributable to known preventable risk factors in Japan. Ann Oncol. 2012;23(5):1362‐1369. 22048150 10.1093/annonc/mdr437

[41] Kato M . がん診療連携拠点病院整備の進捗と第二期への展望 (特集 新たながん対策の推進 : 第二期のがん対策基本計画を踏まえて) Designated cancer hospitals and cancer control in Japan. J Natl Inst Public Health. 2012;61(6):549‐555.

