# Supplementary material for: Is young‐onset esophageal adenocarcinoma increasing in Japan? An analysis of population‐based cancer registries
Source: Cancer Med. 2022 Jan 25;11(5):1347–56. doi: 10.1002/cam4.4528 (PMC8894695; doi:10.1002/cam4.4528)

Supplemental Table 1. Distribution of esophageal cancer cases by histological type, sex and age group, 1993-2014

		Total number of cases	Histological Type (%)			
			Squamous Cell Carcinoma	Adeno-carcinoma	Other types	Unknown/ Missing
**Men**						
All cases		8,986	84.0	3.9	2.0	10.1
Age at diagnosis	<50 years	278	77.3	8.3	3.2	11.2
	50-<60 years	1,376	85.6	4.7	2.7	7.1
	60-<70 years	3,031	87.0	3.7	2.0	7.4
	70-<80 years	3,012	85.4	3.3	2.0	9.4
	80+ years	1,289	73.1	4.2	1.2	21.5
**Women**						
All cases		1,656	75.2	4.7	3.1	17.1
Age at diagnosis	<50 years	58	82.8	3.5	6.9	6.9
	50-<60 years	204	92.2	2.0	2.0	3.9
	60-<70 years	394	85.0	3.6	4.3	7.1
	70-<80 years	466	77.3	4.7	3.9	14.2
	80+ years	534	58.8	6.6	1.5	33.2

Supplemental Table 2. Kaplan-Meier log-rank test for equality comparing the complete data and the imputed data in three cancer registries in Japan[a], by year of diagnosis and histological type

Year	P-value[b]		
	ESCC	EAC	Other types
Men			
1993-1999	0.835	0.957	0.948
2000-2004	0.593	0.961	0.941
2005-2009	0.578	0.708	0.918
2010-2014	0.491	0.812	0.969
Women			
1993-1999	0.407	0.704	0.796
2000-2004	0.471	0.847	0.742
2005-2009	0.819	0.924	0.992
2010-2014	0.684	0.862	0.895

[a] Three Japanese cancer registries, in Yamagata, Fukui and Nagasaki prefectures.

[b] P-values at $p<0.004$ were considered significant after Bonferroni correction.

ESCC: Esophageal squamous cell carcinoma

EAC: Esophageal adenocarcinoma

Supplemental Table 3. Mean annual percent change in the incidence rates of esophageal cancer by histological type and clinical stage at diagnosis, 1993-2014

Age Group	Localized		Regional		Distant	
	Mean APC (%)	95% CI	Mean APC (%)	95% CI	Mean APC (%)	95% CI
**EAC**[1]						
Men						
40-49	14.87	(8.93, 45.89)	-1.63	(-36.34, 7.14)	11.52	(10.32, 20.32)
50-59	10.26	(7.33, 17.62)	0.47	(0.49, 0.94)	0.21	(0.13, 0.28)
60-69	4.74	(3.96, 5.82)	2.11	(1.60, 2.86)	0.23	(-0.12, 0.83)
70-79	2.31	(1.51, 3.53)	0.68	(-0.01, 1.84)	0.96	(0.39, 1.81)
80+	6.07	(3.25, 14.04)	0.97	(-0.43, 3.55)	2.38	(0.62, 6.09)
**ESCC**						
Men						
40-49	1.28	(1.13, 1.4)	-2.81	(-3.07, -2.56)	-2.96	(-3.72, -2.34)
50-59	1.62	(1.60, 1.64)	-1.29	(-1.30, -1.28)	-0.59	(-0.60, -0.58)
60-69	2.23	(2.22, 2.25)	-0.56	(-0.56, -0.56)	0.08	(0.07, 0.09)
70-79	1.71	(1.69, 1.73)	-0.70	(-0.71, -0.7)	-0.68	(-0.70, -0.66)
80+	0.66	(0.60, 0.72)	-0.35	(-0.39, -0.31)	-1.73	(-1.78, -1.69)
Women						
40-49	0.13	(-1.43, 0.84)	2.75	(2.84, 3.30)	-4.98	(-26.52, 1.77)
50-59	2.51	(2.04, 3.21)	0.58	(0.56, 0.62)	0.22	(0.30, 0.45)
60-69	2.38	(2.22, 2.56)	-0.78	(-0.81, -0.75)	-0.74	(-0.80, -0.67)
70-79	-1.00	(-1.01, -0.99)	-2.59	(-2.64, -2.54)	-4.67	(-5.04, -4.35)
80+	-3.10	(-3.16, -3.03)	-3.74	(-3.76, -3.72)	-4.62	(-4.63, -4.61)

[1] APC for women was omitted because of limited number of cases.

APC: Annual Percent Change

EAC: Esophageal Adenocarcinoma

ESCC: Esophageal Squamous Cell Carcinoma

Supplemental Fig. 1. Parameter estimates of cohort and period effects for esophageal adenocarcinoma incidence in men by clinical stage at diagnosis

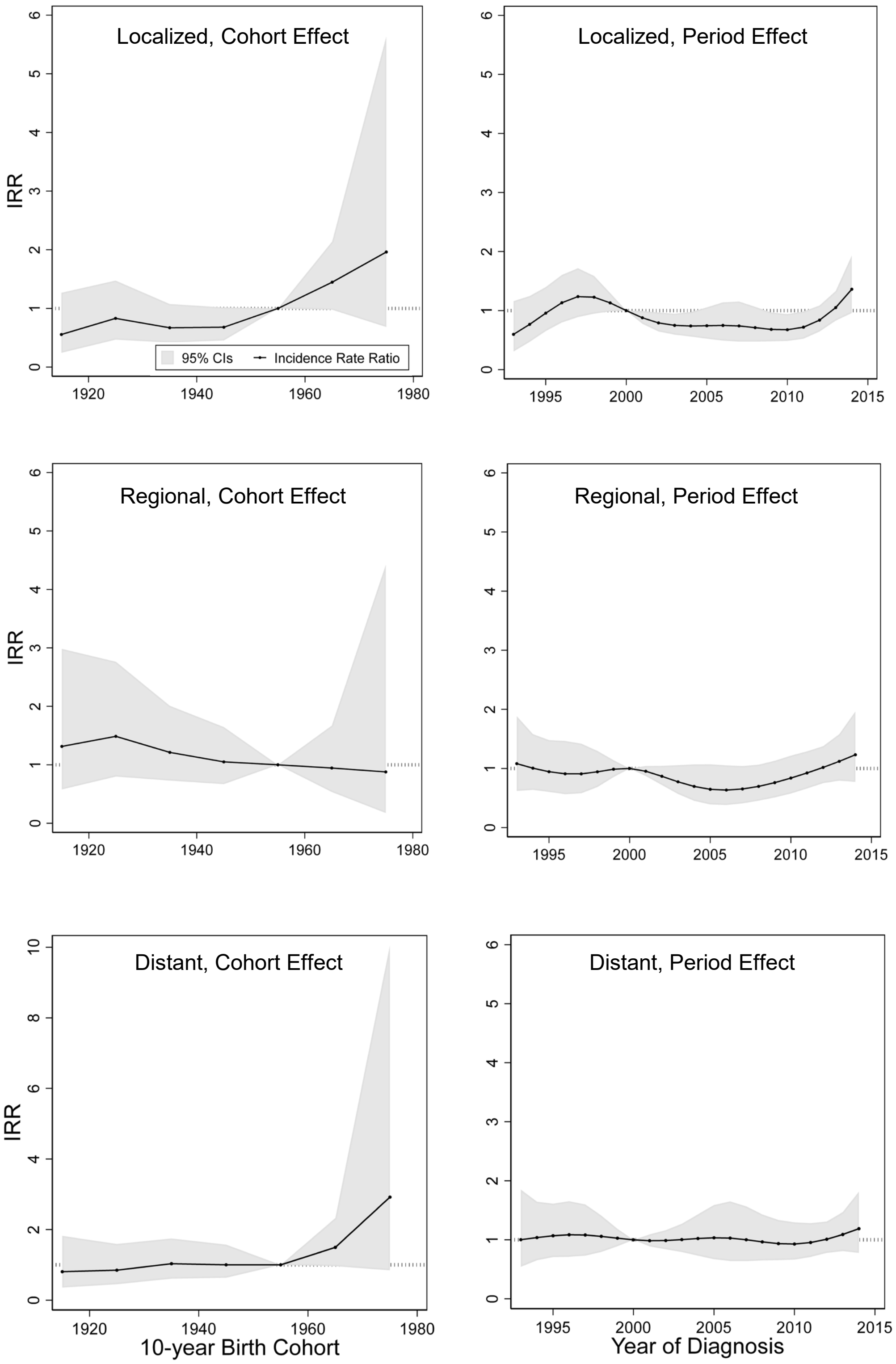

Supplemental Fig. 2. Parameter estimates of cohort and period effects for incidence of esophageal squamous cell carcinoma in men by clinical stage at diagnosis

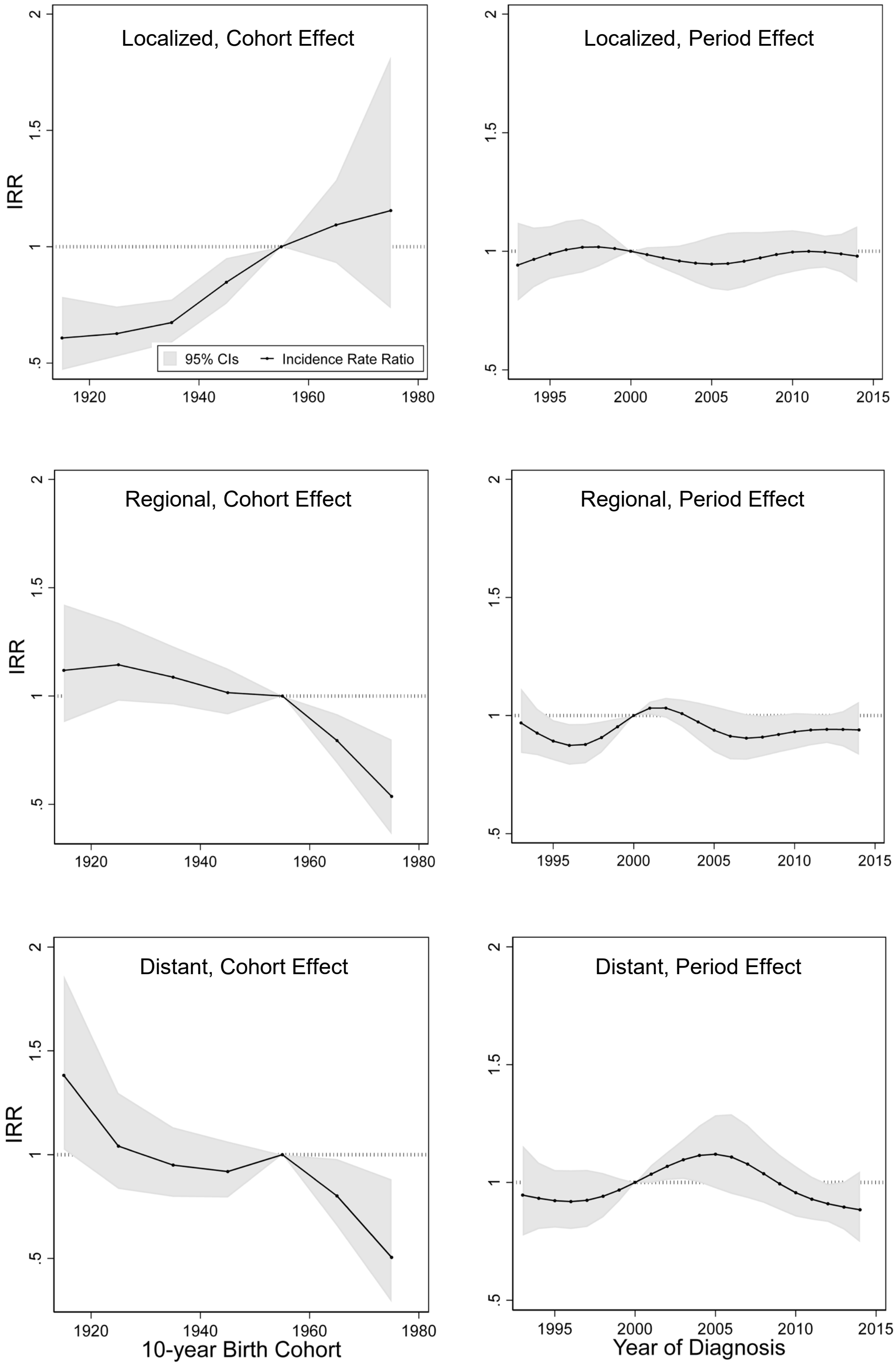

Supplemental Fig. 3. Parameter estimates of cohort and period effects for incidence of esophageal squamous cell carcinoma in women by clinical stage at diagnosis

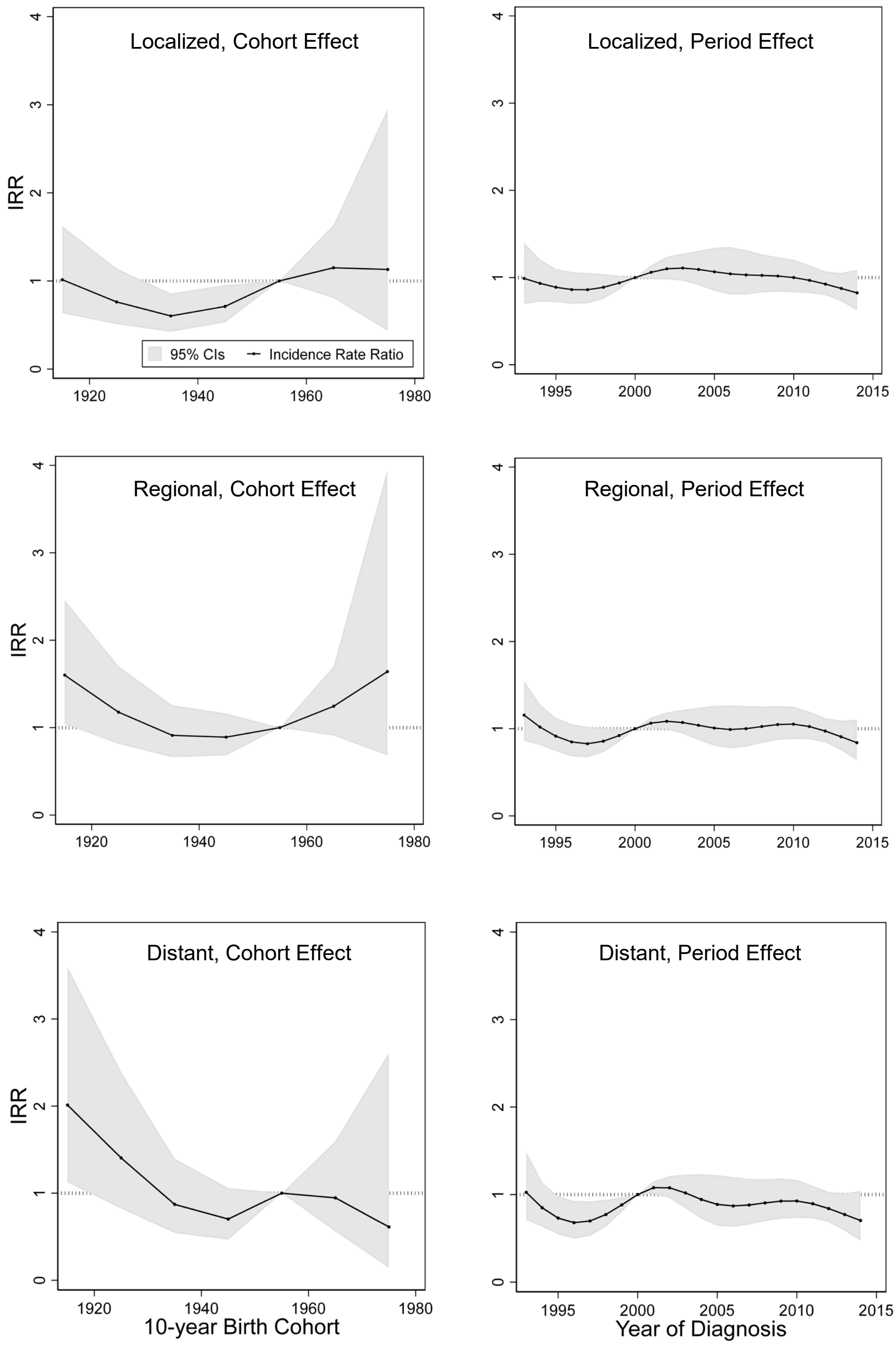

Supplemental Fig. 4. Parameter estimates of cohort effects on the incidence of esophageal cancer by histological type (reference cohort: 1940s)

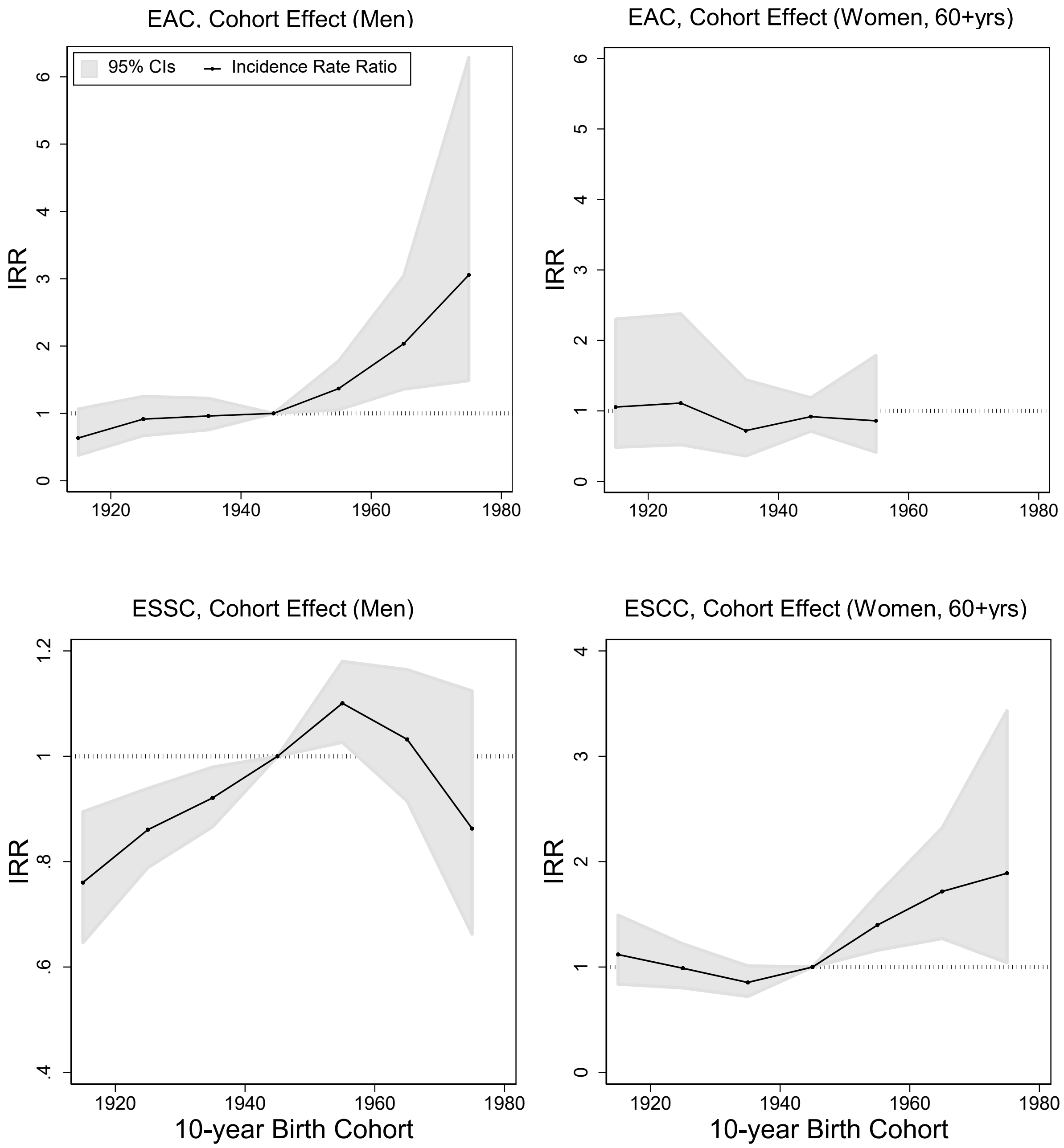

Supplemental Fig. 5. Parameter estimates of cohort and period effects on esophageal cancer incidence in men by histological type before imputation, excluding the "Unknown" histological type

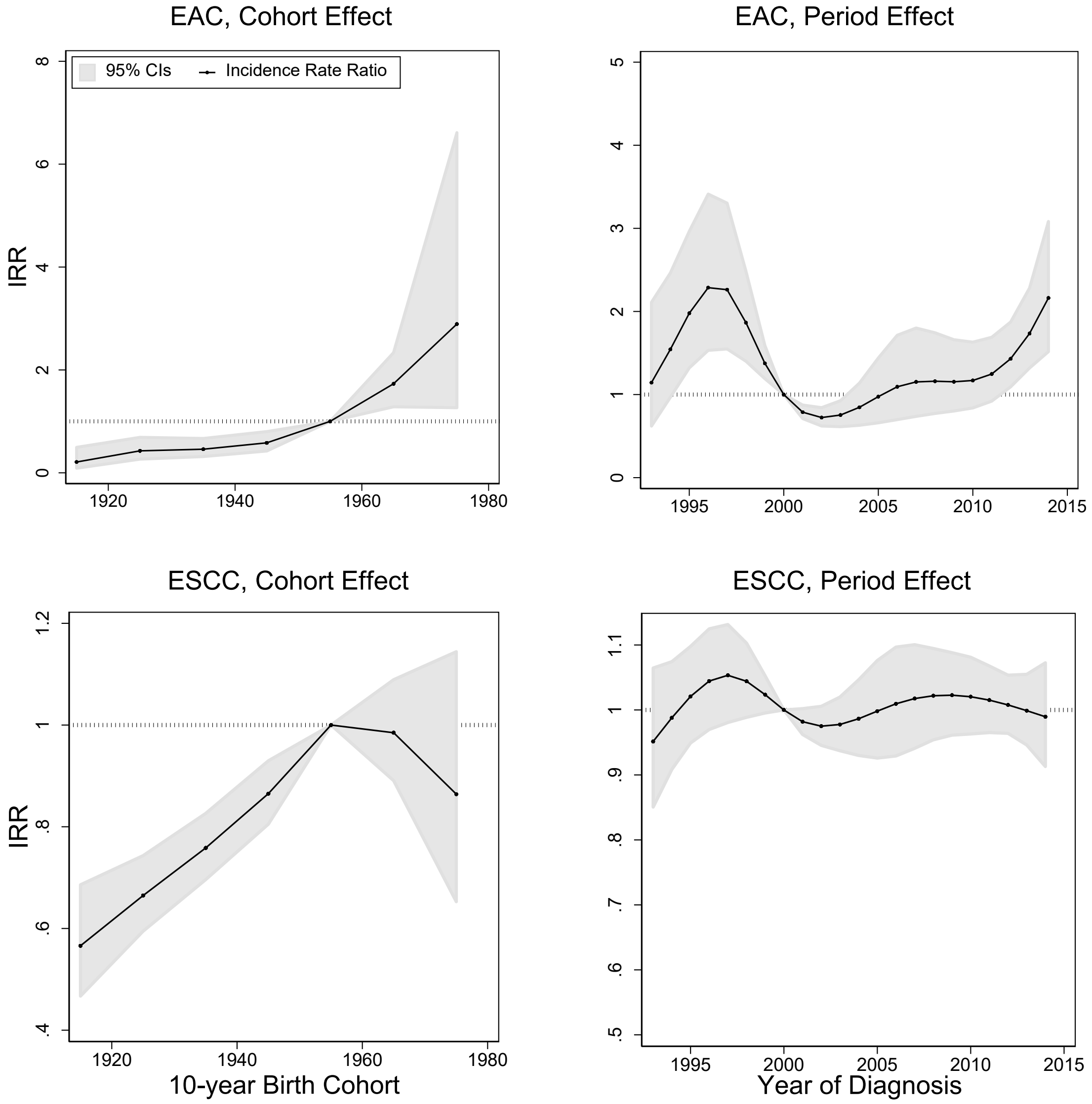

Supplemental Fig. 6. Parameter estimates of cohort and period effects on esophageal cancer incidence in women by histological type before imputation, excluding the "Unknown" histological type

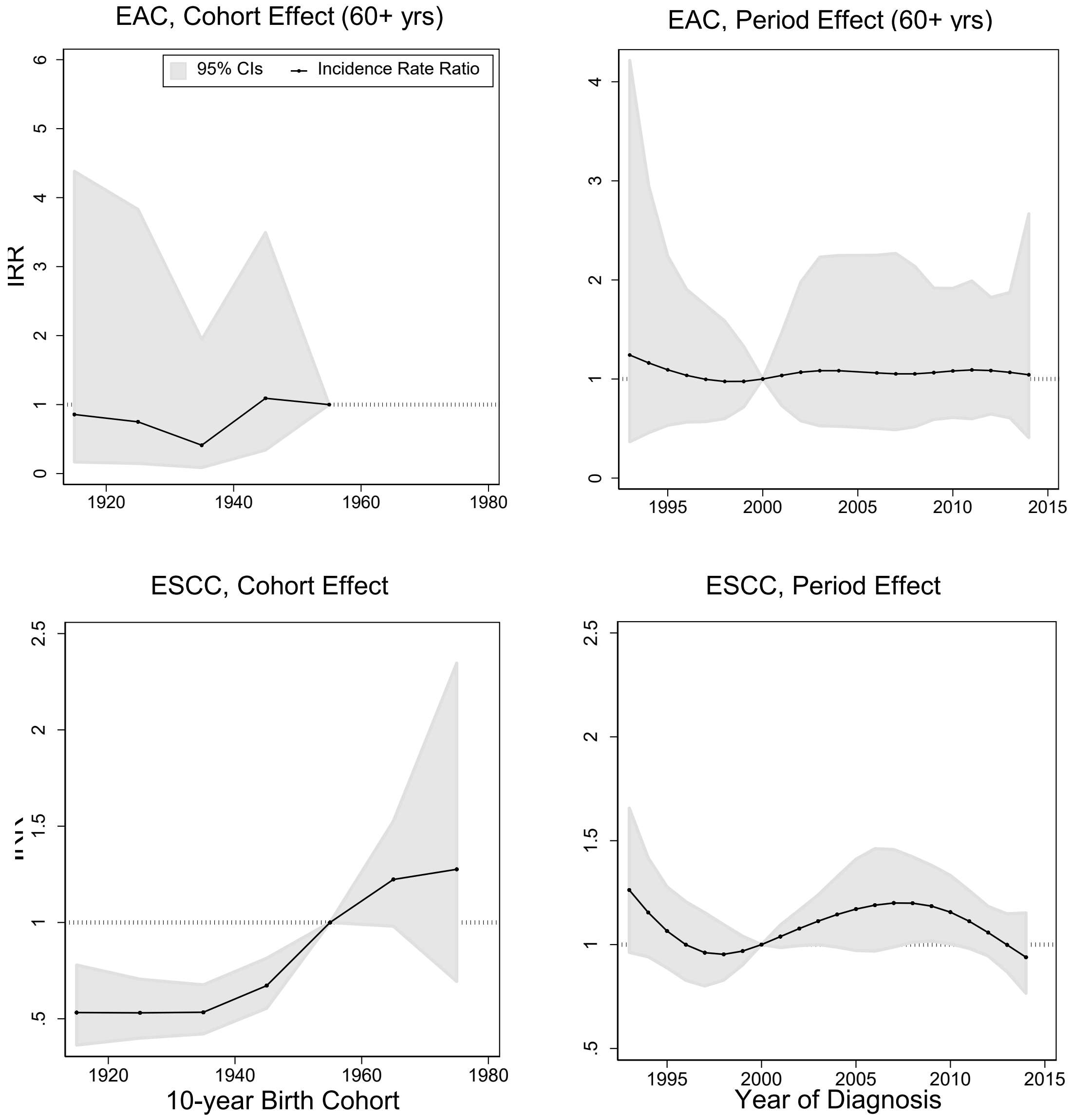

Supplemental Fig. 7. Proportion of current smokers (daily or occasionally) in Yamagata, Fukui and Nagasaki prefectures, 2000-2019

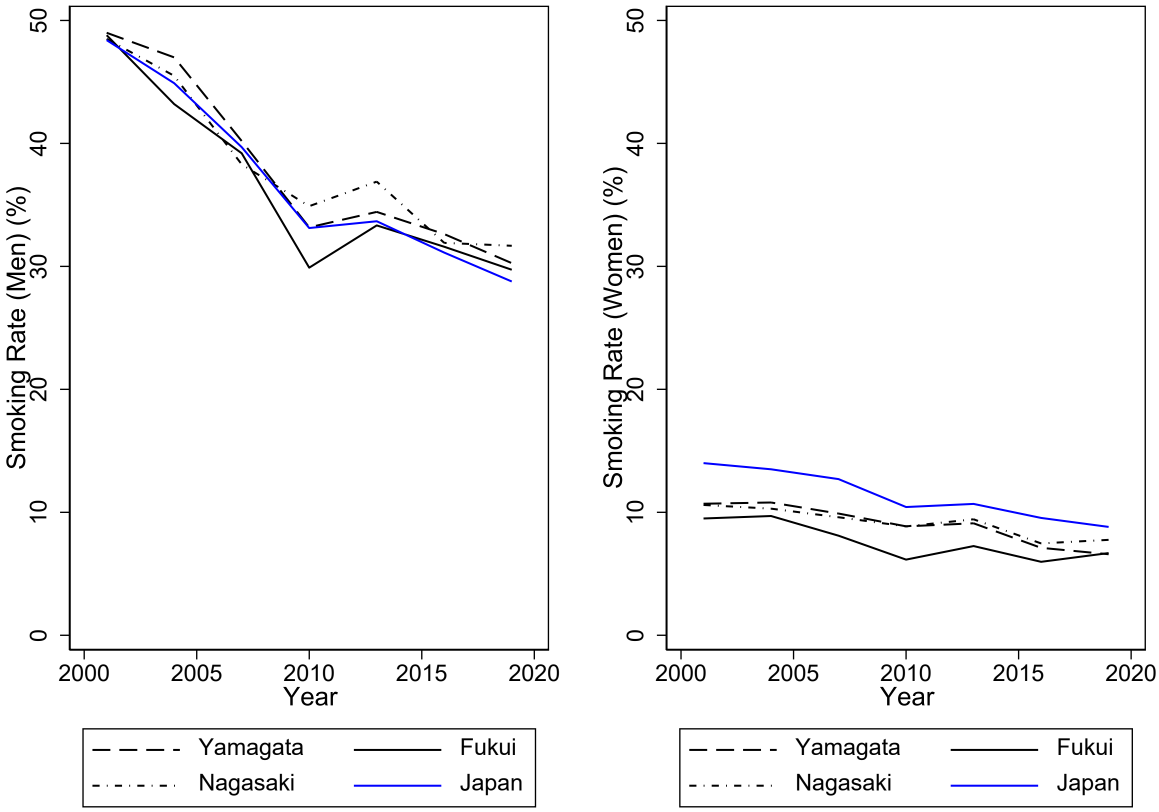

Supplement: Supplementary file 1 — Supplementary Material [file CAM4-11-1347-s001.pdf]
